# Supplementary material for: Genetic Characteristics of Canine Adenovirus Type 2 Detected in Wild Raccoon Dogs (Nyctereutes procyonoides) in Korea (2017–2020)
Source: Vet Sci. 2022 Oct 27;9(11):591. doi: 10.3390/vetsci9110591 (PMC9696381; doi:10.3390/vetsci9110591)
Supplement: Supplementary file 1 [file vetsci-09-00591-s001.zip › vetsci-1972454-supplementary.pdf]

veterinary sciences

MDPI

Supplementary Materials

Supplementary Tables

Table S1. Primers used for PCR and sequencing to obtain the entire gene sequences of the fiber, hexon, penton and pol genes of CAdV-2.

Target gene	Name	Sequence (5′-3′)	Amplicon size (bp)
Fiber	CAdV1_2fiber26100F	AACTGGCAACAAAATCTAGTAACCATA TTTAATCAACACGAG	650
	CAdV1_2fiber26750R	GAAAGTTTAATGGCACCTAACGTAGAAAA	
	CAdV1_2fiber26592F	AGTAAGCGGACACGA AGDKCT	880
	CAdV1_2fiber27656R	CCACTYCCRGCACTTAGAG	
	CAdV1_2fiber27560F	GCGCGGGCTTRTCTRTACA	827
	CAdV1_2fiber28387R	TYATTGATTTTCWCCYACATAGGT	
	CAdV1_2fiber28000F	TTTACTCCACTCCACTCCCGC	500
	CAdV1_2fiber228500R	GTTTTGAATCTGTCGGCAGCGTC	
Hexon	CAdV1_2hexon16650F	CAAACCTGTTTTGGTAGACCGTCC	600
	CAdV1_2hexon17250R	AAGGAGGGGCCTCTGTCTAGG	
	CAdV1_2hexon16936F	ATGGCRACYCCGTCGATGCTG	932
	CAdV1_2hexon17876R	TCTTGCAAGTCCACCACGGCA	
	CAdV1_2hexon17722F	CAGASGRTCAGGTTACAGGKGT	1091
	CAdV1_2hexon18813R	CCACCCTCTRAATCCWGCCCA	
	CAdV1_2hexon18736F	ACCTGTCTKCYGCCAACATGC	883
	CAdV1_2hexon19619R	GAGAAGGGAGTTCTGAGGTAC	
	CAdV1_2hexon19450F	ACTGACCTGGGCCAAAACCTG	550
	CAdV1_2hexon20000R	GCCCTTATACTCAAAGCTGTACACTTGCTTGAG	
Penton	CAdV1_2penton12390F	AGCAGACAAGCTGGCTCGGTGGAAAACATACA	670
	CAdV1_2penton13060R	AGCACCTTGCCAAAGTTGTCTGCTGTGGCG	
	CAdV1_2penton12607F	ATGGAGTTTYCGTCGTCTCC	711
	CAdV1_2penton13319R	TTTTGTAATGCCTAAAAAGTTGTT	
	CAdV1_2penton13173F	GCCTCAAGTTTGACACMAGAA	677
	CAdV1_2penton13850R	TGCTGATAATKGTKGGRGCAGG	
	CAdV1_2penton13691F	AATCCYAGCAGCTACCCAGT	329
	CAdV1_2penton14020R	CTAGAAKGTTTTACTGGACAGC	
	CAdV1_2penton13690F	AATCCYAGCAGCTACCCAGTGGT	610
	CAdV1_2penton14300R	GGGACCGTTGCGCGCCAC	
Pol	CAdV1_2pol4400F	GCAACACTGGGTTATGCTATCTTGAA	600
	CAdV1_2pol5000R	CCCAGAGCTCACTTGGCTAGTAG	
	CAdV1_2pol4642F	TTAAGTCATCCACTGCAGCTCC	855
	CAdV1_2pol5498R	CCGCCTTCTTRCAGCGAAGAA	
	CAdV1_2pol5379F	AAAGTGTGTAAACAAAAGTCATCATC	798
	CAdV1_2pol6177R	TATGACATATGTGGCATGTATGC	
	CAdV1_2pol6027F	TCAATGGTRAAGATGCCYGGAAGA	900
	CAdV1_2pol6927R	GGCAGAAAATMCCACCCGTGT	
	CAdV1_2pol6750F	AAAGTGTCACGCACCATAAATTTTAT	810
	CAdV1_2pol7560R	AATGCAAAAGTGTGAGTTYTGCGG	
	CAdV1_2pol7413F	AGCCTYGSCGGRGCYCCAA	654
	CAdV1_2pol8067R	GTTTCAARGTCACGGGACCAG	
	CAdV1_2pol7600F	AAGACTTTACCTTGCTTAACTAAAAACTTGA	600
	CAdV1_2pol8200R	TAGGGGAGCTAGAGGAGGAGGA	

Table S2. Primers used for PCR and sequencing to obtain the entire genomic sequence of CAdV-2.

Name	Sequence (5′-3′)	Amplicon size(bp)
CAdV2_1F<br>CAdV2_1107R	CATCATCAATAATATACAGGACAAAGAGGT<br>CTGAACGTACAGGCATAGCAAAAG	1106
CAdV2_1040F<br>CAdV2_1900R	AAATGTTTGGGCAGGTTAAATGTTTGGGCA<br>CTTCTTCCGCGGACCCCTGCTCCTCGAGCA	860
CAdV2_1780F<br>CAdV2_2750R	GGCCTTCTGACTCTGTAGTGCCTGAAGCTGACA<br>ACTTCCATTACCCTCGCAAGTGCACAGTTG	970
CAdV2_2600F<br>CAdV2_3525R	TGTGCTTTTGGGGTGGTGACCGGGTCA<br>ATAAATTTTATTTTTACATTAATCTCGGGC	925
CAdV2_3430F<br>CAdV2_4450R	ACATGCTTGCGGTGATGTTCTTTTGGATGAGCT<br>AAGGTCAGTAAGTACCTTAATAAAGATGGAAGC	1020
CAdV2_6350F<br>CAdV2_7300R	CTTTTCTGCTTTATACAAGATTTGTTTAAAAATGGC<br>TTGCCAAAACTCTTGCAACAGAGCAAAACTGGG	950
CAdV2_7150F<br>CAdV2_8050R	GGGGATTTTGGCAAATCACGTGGGACCAGAGGC<br>AACCGCCAAATCCAATCAATCAACAGGAGA	900
CAdV2_7950F<br>CAdV2_8935R	TTGCGCTTGGGTGGGTTTACTATGGTGGAAGTT<br>TCTTTACCAGCCAATCTGTAACAAAAAGCATTATT	985
CAdV2_10804F<br>CAdV2_11750R	GTGCTAATATTAGGTCCAAAGAGGAGTGGGGGG<br>TGCTCACCTCTGTGACCTCTTCAAGAGTACGCT	946
CAdV2_11650F<br>CAdV2_12500R	GTGTCTCCCTTTACAGACAGTGTTAGCATTAGC<br>GCCTGCTCATCCCATCATCACTGTCATCGCTGG	850
CAdV2_12390F<br>CAdV2_13060R	AGCAGACAAGCTGGCTCGGTGGAAAACATACAA<br>AGCACCTTGCCAAAGTTGTCTGCTGTGGCG	670
CAdV2_14600F<br>CAdV2_15500R	CGCCATGGCAGCAATCAGTCGCGCCATCAAGCA<br>CGGTTCTGGCGGCAGCGGTAGCTGGGGTGGCAG	900
CAdV2_15380F<br>CAdV2_16200R	AGCCCAGCTCTTTTTTTGAGGTGCGCTCTC<br>TCATAGCCTGGGCCCCGTAGCGTGGTGCCAAGA	820
CAdV2_16050F<br>CAdV2_16920R	TCATCCCTTTGATAGCTGCCGCCATTGGGGCAG<br>TCACTTGAGTATATTTAATCACAGTTAGAAGCA	870
CAdV2_18520F<br>CAdV2_19300R	GCTATTGCTCGTTCCATATTCAGGTCCCTCAAAAAT<br>TGGTAGGCAGCATAGTTGGCCACGTCCACC	780
CAdV2_19150F<br>CAdV2_20000R	TGTGGCCCAGTCCAACATGACAAAAGACTGGTTCAT GGT<br>GCCCTTATACTCAAAGCTGTACACTTGCTTGAG	850
CAdV2_19850F<br>CAdV2_20740R	CCGGGTTTCATCTCTTCACAAAGAATGGCCTGC<br>GGCAGGCAAACTTGCAAGATTACCCCATTCTCT	890
CAdV2_20600F<br>CAdV2_21450R	CAATTTTTTTGGGGGGCGGCCTTGCTGTTG<br>GGCTAGTGGCCTCTAAGTTTTTTAAAGCTA	850
CAdV2_21300F<br>CAdV2_22020R	TTTTAGAGGTGGTAATGTAAGCCTGGACTG<br>CGTTCGTAGGCGCCAGCTTAGCTCTGATACA	720
CAdV2_23150F<br>CAdV2_24000R	GTCCGGCATCCTGCCCTCCATGACCTGCGCGCT<br>GTTGTTGGGAAGATTAACTCCCTCGCGAAAGAG	850
CAdV2_23850F<br>CAdV2_24720R	TCAGAGGAACGAGCTCCTAGATGGGACCAAAAA<br>ATGCCCGTGCCAGCCAGCACTGGGCTTTTTATA	870
CAdV2_24600F<br>CAdV2_25500R	TCAGGAAGCCGCCCCACCCAAAACGGTCACTCT<br>CACTTCTAGGTGTAGGTGTAGGAGGCAAGGTGT	900
CAdV2_25350F<br>CAdV2_26210R	AGGCAGCCTGTTCTATGTATAACCTTAACTAGA<br>ATTGACTTGGCTCTGCAAGTTACTCTAAATAAGCCT	860
CAdV2_26100F<br>CAdV2_27110R	AACTGGCAACAAAATCTAGTAACCATATTTAATCAACACGAG<br>AGTACCACCTGTTTTTGTGAGGGGAGGGGAGAATTGTAG	1010
CAdV2_28220F<br>CAdV2_29180R	ACTTACACCCTTACCTTTAGGTTTTTAAACTTT AAC<br>GGGTTTACATGCAATGCGATTGAGTTGAGTTGA	960
CAdV2_29050F	TTAATAAAGTCCTCAATCACTTTCTGAACCACATGC	700

CAdV2_29750R	GCGGAGCTAGCTTTGTTTACTGTAAAATGTGTTTCA	
CAdV2_29600F	ATAGAAGCAGTAACATCATCAATGGTGTCGAAGAGA	700
CAdV2_30300R	GCGGGCCCCTCATTGTGACTTCTATTGTGTATACTA	

Table S3. Primers used for PCR and sequencing to obtain the genomic sequence of CAdV-2 via 5' and 3' Rapid Amplification of cDNA Ends (RACE).

Target	Name	Sequence (5′-3′)	Primer sequence location(bp)
3′RACE	3RACE_POLY_A	GGCCACGCGTCGACTAGTAC(T)17	-
	3RACE_adaptor	CCGGTGCGCAGCTGATCATG	-
	3RACE_GSP1	AAAGGTAGGACACGGACGCACT	30690-30711
5′RACE	5RACE_GSP_P	*GTGGAATAGGTGAAACCACGGAGT	750-773
	5RACE_GSP1	ACA GCC CTT TTG TGG GCC TGT G	660-681
	5RACE_GSP2	ATTGATTTGCGGTTTAGCGGGTGG	200-224

* Phosphorylation.

Supplementary Figures

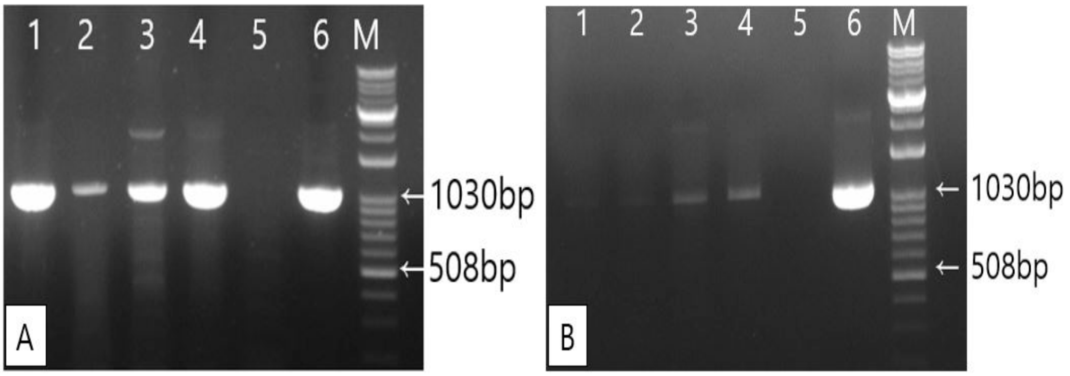

Figure S1. Agarose gel images corresponding to each tissue type from the two carcasses that tested positive in the PCR investigation. Corresponds to 18Ra-54 (A) and to 18Ra-65 (B) strain; starting in order from lane 1: liver, kidney, lung, intestine, negative control, positive control, and marker. The PCR amplification size is 1030 bp.

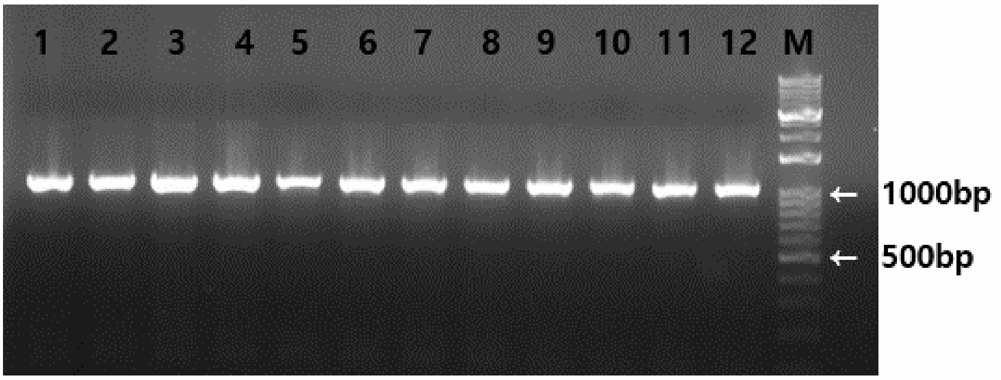

Figure S2. The Vero cells inoculated with the 18Ra-54 strain were cultured in media. DNA of cells from each passage were amplified and presence of 18Ra-54 strain was confirmed using PCR. In order from lanes 1 to 12: DNA corresponding to cells isolated from the media of virus passage 1–6. Lane 1 is the media from passage 1, lane 2 is the cell lysate from passage 1, lane 3 is the media from passage 2, lane 4 is the cell lysate from passage 2, and lane 12 is the cell lysate from passage 6 in this order. The PCR amplification size is 1030 bp.

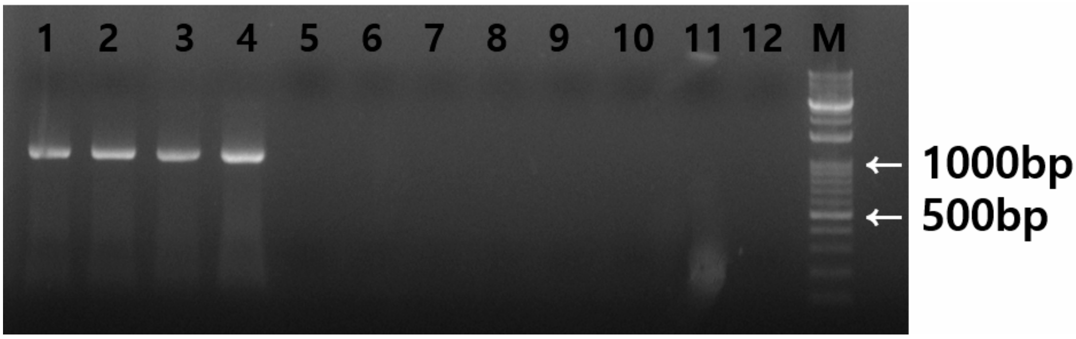

Figure S3. The Vero cells inoculated with the 18Ra-65 strain were cultured in media, and DNA of cells from each passage were amplified and presence or absence of 18Ra-65 strain confirmed using PCR. In order from lanes 1 to 12: DNA corresponding cells isolated from media of virus passage 1–6. Lane 1 is the media from passage 1, lane 2 is the cell lysate from passage 1, lane 3 is the media from passage 2, lane 4 is the cell lysate from passage 2, and lane 12 is the cell lysate from passage 6 in this order. The PCR amplification size is 1030 bp.
